# The use of monoclonal antibody therapy in pediatric patients with COVID-19: a retrospective case series

**DOI:** 10.1186/s12245-022-00414-8

**Published:** 2022-03-03

**Authors:** Jesse De Los Santos, Donna Bhisitkul, Matthew Carman, Kayla Wilson, Shannon Hasara, Karen Homa, Pedro Reyes, Andrew Bugajski, Andrew Barbera

**Affiliations:** 1 Department of Emergency Medicine, Lakeland Regional Health, 1324 Lakeland Hills Blvd, Lakeland, FL 33805 USA; 2 Department of Research and Sponsored Studies, Lakeland Regional Health, 1324 Lakeland Hills Blvd, Lakeland, FL 33805 USA

**Keywords:** COVID-19, Bamlanivimab, Casirivimab, Imdevimab, Pediatric

## Abstract

**Background:**

Monoclonal antibody (MCA) therapies have been utilized under emergency use authorization (EUA) for high-risk pediatric patients with mild to moderate coronavirus disease 2019 (COVID-19) in the outpatient setting since late 2019. The purpose of this study was to describe the use of MCA therapy in pediatric patients in the pediatric emergency department (ED) at a large community hospital.

**Methods:**

This was a retrospective case series of high-risk pediatric patients 12 to 17 years of age who received MCA therapy in the pediatric ED between December 8, 2020 and June 3, 2021. The primary outcome was to describe the patient characteristics, clinical presentation, and safety profile of the pediatric population that received MCA therapy. The secondary outcome was to describe the incidence of hospitalizations or ED visits up to 28 days following therapy.

**Results:**

A total of 44 patients were included in the analysis. The median number of days of symptoms was 4 with 41% of patients having symptoms between 0 and 3 days at time of MCA administration. Only one patient experienced a mild adverse event that did not require epinephrine administration. Two patients returned to the ED for reevaluation during the study follow-up period. No patients required admission within 28 days post-therapy.

**Conclusions:**

The administration of MCA therapy in high-risk pediatric patients in the pediatric ED was well-tolerated with subjective improvement noted in COVID-19 symptoms post-therapy. Further studies are necessary to determine the role MCA therapy may play in reducing morbidity from COVID-19 infection in high-risk pediatric patients.

## Introduction

### Background

In December 2019, a severe acute respiratory syndrome-associated virus (SARS-CoV-2; COVID-19), currently responsible for a global health disease pandemic, was discovered in Wuhan, China [1]. As of September 2, 2021, a total of 39 million confirmed cases and 638,000 deaths attributed to COVID-19 have been reported in the USA alone [2]. Patients with COVID-19 may present clinically with a variety of symptoms, including fever, respiratory complications, gastrointestinal disturbances, fatigue, and neurological syndromes as well as anosmia and ageusia [3, 4].

A recent literature review has shown a positive correlation between advanced age and increased morbidity and mortality in patients with COVID-19 disease. Additional co-morbidities such as cardiovascular disease, diabetes, respiratory disorders, and immunocompromising conditions may lend to more severe clinical manifestations and increased risk of death in adults [5]. In children, however, COVID-19 infection occurs in less than 12% of all diagnosed cases. Children with pre-existing health conditions, such as obesity, may be at a higher risk of clinical decompensation and serious illness [6]. Although rare, when serious complications arise in children, they are diverse and span multiple organ systems. Children with no previous cardiac history who contract COVID-19 are at higher risk for fulminant myocarditis, ventricular arrhythmias, and pulmonary hypertension [7]. Neurological findings including status epilepticus, encephalitis, Guillain-Barré syndrome, acute demyelinating syndrome, and psychosis have also been reported in this patient population [8]. A rare, but serious complication of COVID-19 includes multi-system inflammatory syndrome in children (MIS-C), however, literature suggests the epidemiology of MIS-C differs from severe COVID-19 disease [9–11]. Although serious complications or death from COVID-19 remain uncommon in pediatrics, 10% of all cases were deemed severe and there is still a dearth of evidence regarding the optimal treatment for mild to moderate COVID-19 in this population [12, 13].

Monoclonal antibody (MCA) therapy targeted against the spike protein of the SARS-CoV-2 virus was granted Emergency Use Authorization (EUA) by the Food and Drug Administration (FDA) for mild to moderate COVID-19 infections in high-risk individuals who were at least 12 years of age and weighing at least 40 kg [14]. Specifically, MCA therapy is used to target viral entry, promote passive immunity, and minimize overall viral load in infected patients [15, 16]. The first MCA therapy authorized by the FDA was bamlanivimab on November 9, 2020 [14]. Additional MCA therapies including REGN-COV2 (casirivimab/imdevimab) and bamlanivimab/etesevimab were also granted EUA on November 21, 2020 and February 9, 2021, respectively [17, 18]. At the time of this writing, the National Institutes of Health cite insufficient evidence to recommend for or against administration of MCA therapy in pediatric patients [19]. There is a paucity of data on the efficacy and tolerability of MCA therapy in pediatric patients as little research has been conducted in this patient population. Mak and colleagues recently published their experience using MCA therapy in 17 pediatric patients and reported that treatment was well-tolerated and may be effective in limiting progression to more severe disease, though this is the first preliminary report published in this area [20].

Given the large gap in the literature regarding efficacy of MCA therapy in pediatrics, the purpose of this study was to describe the use of MCA therapy in pediatric patients in the pediatric emergency department (ED) at a large community hospital.

## Materials and methods

### Study design and setting

This was an institutional review board-approved (ID#1771910-2), retrospective, observational study of high-risk pediatric COVID-19 positive patients who received MCA therapy in the pediatric ED. This study was conducted in an 864-bed community hospital in the southern USA which houses a 33-bed pediatric ED with a pre-pandemic volume of over 50,000 annual visits. The study institution was designated as an MCA treatment site by the Department of Health due to its capacity to identify eligible pediatric patients, administer MCA therapy with necessary expertise and equipment to identify and treat any adverse infusion reactions, and to deliver evidence-based care to patients with COVID-19 infections. Additionally, COVID-19 testing was readily available along with notification to parents and guardians of the COVID-19 results [21].

### Selection of participants

An analytics report designed to query the study population was generated from the electronic health record (EHR). Once patients were identified for inclusion, two study team members reviewed the medical records for accuracy. Upon confirmation of patient inclusion/accuracy, data points were recorded per patient. Data points included patient demographics (including age and gender), body mass index (BMI), comorbidities, symptoms at the time of presentation, duration of symptoms, and corticosteroid therapy within the previous 7 days. Clinical characteristics included vital signs (blood pressure, oxygen saturation, heart rate, respiratory rate, and temperature) at the time of infusion. Patient outcomes were any infusion-related events (including but not limited to difficulty breathing, reduced oxygen saturation, chest pain, and hypotension), and hospitalization or ED visit within 28 days. Abnormal vital signs were defined as temperature 38 °C and greater, heart rate 120 beats per minute and greater, and respiratory rate of 24 breaths per minute and greater. Oxygen saturation was considered abnormal if less than 95% on room air.

Inclusion and exclusion criteria were based on MCA therapy administration guidelines from the EUA [14, 17, 18]. Patients were included in this study if they tested positive for and had symptoms of COVID-19, were 12 to 17 years of age and weighed greater than 40 kg. It is important to note that participants were only eligible to receive MCA therapy if they met at least one high-risk criteria listed in the MCA therapy EUA (Table 1). All patients included in this study were treated in the ED between December 8, 2020 and June 3, 2021. Patients were excluded if they required hospitalization, were treated with oxygen therapy or had an increase in baseline oxygen therapy requirements, or had COVID-19 symptoms for greater than 10 days. These exclusions were based on the limitations of authorized use listed in the EUA [14, 17].

**Table 1:** High-risk criteria for treatment of mild to moderate COVID-19 in pediatric patients

• Body mass index (BMI) ≥ 85th percentile	
• Chronic kidney disease	
• Diabetes	
• Immunosuppressive disease or Immunosuppressive therapy	
• Chronic lung disease (e.g. chronic obstructive pulmonary disease, moderate to severe asthma, interstitial lung disease, cystic fibrosis, pulmonary hypertension)	
• Neurodevelopmental disorders	
• Cardiovascular disease (including congenital heart disease) or hypertension	
• Medical-related technological dependence	

### Interventions

#### Determination of eligibility for MCA administration

**Fig. 1:**
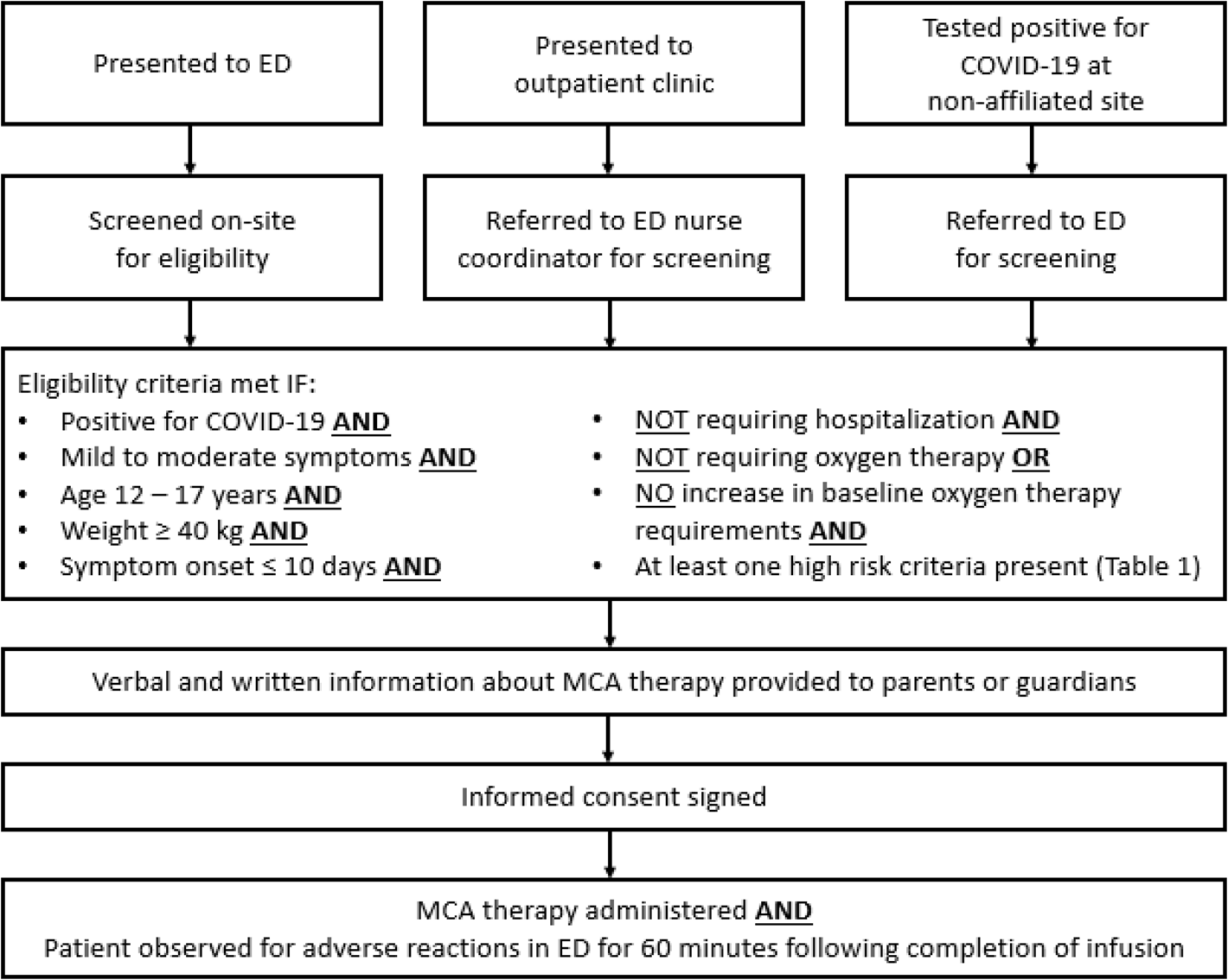
Pathways to MCA administration

Patients that met the criteria presented for MCA administration via three distinct pathways (Fig. 1). These pathways were well documented thus allowing filtering of the EHR data to identify study participants. The pathways were (1) patients who presented to the ED and were screened for MCA therapy on-site; (2) patients who were first seen at an outpatient clinic and then referred to an ED nurse coordinator for screening for MCA therapy; or (3) patients who tested positive for COVID-19 at non-affiliated sites, were referred to the ED, and then screened for MCA therapy. In each pathway, if the inclusion criteria were met, the parents or guardians were given verbal and written information about MCA therapy and completed an informed consent. Upon MCA administration, patients were observed in the ED for 60 min after the infusion for any adverse reactions, in accordance with the EUA administration requirements.

#### Follow-up phone calls

Per institutional standard of care, an ED physician telephoned the parent or guardian 30 days after the ED visit to ascertain whether the MCA therapy subjectively improved the patient’s symptoms, and if improvement was noted, when this improvement was first noticed (days). The caretaker was asked whether the patient had an ED visit related to COVID-19 symptoms at another community facility, if symptoms became worse, how long these symptoms lasted, and if they would choose MCA therapy again, if needed. Three follow-up attempts via telephone were made on a monthly basis between 1 month and 4 months post-infusion.

### Outcomes

The primary outcome was to describe the patient characteristics, clinical presentation, and safety profile of the pediatric population that received MCA therapy. The secondary outcome was to describe the incidence of hospitalizations or ED visits up to 28 days following therapy.

### Analysis

Descriptive statistics were reported as medians and interquartile ranges for continuous variables, and frequencies and percentages for categorical variables.

## Results

### Characteristics of study subjects

Between December 8, 2020 and June 3, 2021, 315 patients aged 12 to 17 years old tested positive for COVID-19 at the study institution and 83 patients met inclusion criteria for MCA therapy. Of these, 44 pediatric patients proceeded to receive MCA therapy. Twenty-four patients (56%) received bamlanivimab, 10 patients (23%) received bamlanivimab/etesevimab, and 10 patients (23%) received casirivimab/imdevimab (REGN-COV2).

### Main results

There were slightly more females than males (57% versus 43%), and 52% of patients were Caucasian (Table 2). The ages ranged from 12 to 17 years with the median age being 15 years. Forty-one percent of participants were between the ages of 16 and 17 years old. The median number of days of symptoms was four with 41% of patients having symptoms between 0 and 3 days. Eighty percent of patients were obese and nearly 43% had asthma. Fourteen percent (*n* = 6) reported steroid use in the previous 7 days. The median oxygen saturation was 98% with two patients having an oxygen saturation of less than 95%. The patients median heart rate was 89 bpm with 7% of the patients having a heart rate of 120 bpm and greater. One patient had a fever with a body temperature of 38.1 °C.

**Table 2:** Summary of patient characteristics, clinical indicators, and patient outcomes

	*N* = 44 *n* (%)
**Monoclonal antibody therapy**	
Bamlanivimab	24 (56)
Bamlanivimab/etesevimab	10 (23)
Casirivmab/imdevimab	10 (23)
**Age (years)**	
median (interquartile range)	15 (14–16)
12 to 13	9 (21)
14 to 15	17 (39)
16 to 17	18 (42)
**Gender**	
Female	25 (57)
Male	19 (43)
**Race**	
Caucasian	23 (52)
Hispanic	11 (25)
Black	10 (23)
**Days of symptoms before MCA**	
Median (interquartile range)	4 (2-7)
0 to 3	18 (41)
4 to 5	11 (25)
6 to 9	15 (34)
**Risk factors** ***(patient may have more ≥ 1 factor)***	
Obesity	35 (80)
Asthma	19 (43)
Developmental delay	2 (4.5)
Hypertension	1 (2.3)
Chest pain	1 (2.3)
Congenital heart disease	1 (2.3)
Insulin dependent diabetes mellitus	1 (2.3)
**Body mass index**	
> 85th percentile	35 (80)
**Steroid use**	
Last 7 days	6 (14)
**Initial oxygen saturation (%)**	
Median (interquartile range)	98 (98–99)
Normal, ≥ 95	42 (95)
Abnormal, < 95	2 (4.5)
**Initial heart rate (bpm)**	
Median (interquartile range)	89 (79–104)
Normal, < 120	41 (93)
Abnormal, > 120	3 (6.8)
**Initial respiratory rate (breaths per minute)**	
Median (interquartile range)	20 (18–20)
Normal, < 24	41 (93)
Abnormal, ≥ 24	3 (6.8)
**Body temperature (celsius)**	
Median (interquartile range)	36.9° (36.7°–37.4°)
Normal, < 38 °C	43 (98)
Abnormal, ≥ 38 °C	1 (2.3)
**Clinical outcomes**	
Infusion-related event	1 (2.3)
ED visit within 28 days	2 (4.7)
Hospitalization within 28 days	0 (0)

*MCA* monoclonal antibody, *bpm* breaths per minute, *ED* emergency department

One patient experienced shortness of breath and flushing within five minutes of infusion initiation. The therapy was immediately stopped and diphenhydramine and dexamethasone were administered. The patient’s guardian attributed this reaction to the patient’s past medical history of anxiety.

None of the patients were hospitalized within 28 days and two patients returned to the ED within 28 days. One patient returned to the study institution the next day with shortness of breath and was treated with albuterol and discharged home. The other patient returned to the study institution 2 days later due to cervical lymphadenitis, which was treated with amoxicillin and discharged home.

Phone follow-up was attempted for 43 out of the 44 patients. One patient was not included, as they did not complete therapy due to a potential infusion-related reaction described above. Forty parents and guardians (93%) were contacted by phone 1 month after the patient’s ED discharge. Three parents and guardians were not available for follow-up during the study time period. Thirty-eight out of the 40 parents and guardians (95%) answered “yes” to improvement after MCA therapy. One parent or guardian stated “no” improvement, adding that the patient experienced “…a migraine during the infusion and felt worse for a few days after.” The other patient came back to ED reporting shortness of breath. Twenty-three out of 38 parents and guardians (61%) reported improvement within 1 day, 13 (34%) reported improved in 2 days, one (3%) reported improvement in 3 days, and one parent or guardian reported that the patient felt worse for 5 days, however, they stated, “…it wasn't related to COVID-19 or the infusion.” Most of the parents and guardians reporting improvement of the patient’s symptoms had positive comments about their MCA therapy, including that the patient “…was better faster than family members who did not get MCA,” “…got better after one day while dad, mom, and sister did not get MCA and all got very sick,” and “…immediately better.” Forty out of 41 (98%) parents/guardians stated they would do MCA therapy again.

## Discussion

MCA therapy continues to evolve over the course of the global COVID-19 pandemic. At the time of this writing, a multidisciplinary panel of expert pediatric infectious disease specialists have published interim guidance suggesting against the routine administration of MCA therapy to pediatric patients, citing the lack of supporting safety and efficacy in children and overall milder clinical disease course [22]. The purpose of this study was to bridge the gap in literature by describing the use of MCA therapy in an outpatient, high-risk pediatric patient population. Whether or not this paucity stems from a lack of access to MCA therapy or an overall milder disease burden in the pediatric population, the need to further describe MCA efficacy and safety in this age group was preliminarily addressed in this study.

Overall, the study results suggest that MCA therapy was well-tolerated and prevented further clinical decompensation in pediatric patients, which is consistent with early reports from the adult population [14, 17, 18]. In this sample of 44 patients, obesity was the most prevalent risk factor occurring in 80% of study patients. This finding was anticipated as the Centers for Disease Control and Prevention (CDC) reports the prevalence of childhood obesity at nearly 19.3% [23]. Obesity may be the biggest risk factor driving MCA administration given the increasing prevalence of obesity in this population. Furthermore, only one patient experienced an adverse event during MCA therapy in this study. This patient experienced flushing followed by mild shortness of breath, which are both previously reported with MCA therapy in adults, though the patient’s history of anxiety may have contributed to this event [14, 17, 18]. Additionally, there were only two ED follow-up visits for mild symptoms and there were no reported hospitalizations during the study time frame.

With a lower complication rate of COVID-19 and milder disease course in the pediatric population, establishing a risk profile for MCA therapy should be paramount for future research. Most of the patients in this study seemed to have an improvement of symptoms within days with some reporting feeling “better after one day.” Whether this was due to the MCA treatment itself or the naturally milder course of disease in this population remains unknown. Additional research is warranted to evaluate the use of MCA therapy in preventing other serious complications or decompensation in children, including MIS-C. However, if widespread vaccination in this age group becomes more prevalent, the number of patients presenting clinically with mild to moderate disease may be expected to decrease, thus limiting future studies from obtaining an adequate sample size to fully ascertain the safety and efficacy of MCA therapy.

The impact of viral mutation and the emergence of variants on the efficacy of MCA therapy remains to be fully elucidated. In May 2021, distribution of bamlanivimab and bamlanivimab/etesevimab to a number of states was halted following guidance from the CDC after the agency identified that the combined frequency of the P.1 and B.1.351 variants now exceeded 10% in those states. Data from in vitro studies suggested that these variants were not susceptible to bamlanivimab or the combination product. As a result, the study institution was required to use casirivimab/imdevimab exclusively as available in vitro data indicated that this product was likely to retain activity against the P.1 and B.1.351 variants [24]. At the time of this writing, the delta variant has swept across the USA, becoming the predominant variant responsible for over 99% of new COVID-19 cases [25]. Along with an increase in transmissibility, early data from a large UK study revealed a more than 30% reduced vaccine effectiveness against the delta variant compared to the earlier, alpha variant [26]. In an already high-risk population, it is reassuring that MCA therapy with casirivimab/imdevimab retains efficacy against the delta variant while reducing disease burden and clinical decompensation [18].

There are several notable limitations to this study. This was a retrospective chart review, which relied heavily on documentation noted within the institution’s EHR. Not all patients that qualified agreed to receive MCA therapy so the study could be biased by the process of enrollment. The study does not have a control group; therefore, outcomes of patients who did not receive treatment are unknown. Additionally, the survey telephone follow-up calls relied on the subjective reporting from the parent or guardian of the patient’s symptom improvement or other clinical outcomes, and not an evaluation made by an objective third party. This process did, however, allow study personnel to ascertain whether patients presented to another facility for symptom management or progressive worsening after the infusion. Finally, similar to the recently published brief report from Mak et al., there are few potential confounders including evolvement of SARS-CoV-2 virus strain mutations and the variability of use with several different MCA therapy products during the study time frame [20].

## Conclusions

In summary, this retrospective review adds to the preliminary data on MCA therapy in high-risk pediatric patients. Although MCA therapy was well-tolerated and appeared efficacious in this sample, further research on a larger scale is needed to rigorously evaluate the use of MCA therapy in children.

## Data Availability

The datasets generated and/or analyzed during the current study are not publicly available but are available from the corresponding author on reasonable request.

## Abbreviations

BMI: Body mass index
CDC: Centers for Disease Control and Prevention
COVID-19: Coronavirus disease 2019
EHR: Electronic health record
ED: Emergency department
EUA: Emergency use authorization
FDA: Food and Drug Administration
MCA: Monoclonal antibody
MIS-C: Multisystem inflammatory syndrome in children
REGN-COV2: Casirivimab/imdevimab
